# Predicting Disease Progression and Mortality in Aortic Stenosis: A Systematic Review of Imaging Biomarkers and Meta-Analysis

**DOI:** 10.3389/fcvm.2018.00112

**Published:** 2018-08-22

**Authors:** Alain Nchimi, John E. Dibato, Laurent Davin, Laurent Schoysman, Cécile Oury, Patrizio Lancellotti

**Affiliations:** ^1^GIGA Cardiovascular Sciences, Department of Cardiology, Heart Valve Clinic, CHU Sart Tilman, University of Liège Hospital, Liège, Belgium; ^2^Department of Medical Imaging, Centre Hospitalier de Luxembourg, Liège, Luxembourg; ^3^Department of Medical Imaging, CHU Sart Tilman, Liège, Belgium; ^4^Gruppo Villa Maria Care and Research, Anthea Hospital, Bari, Italy

**Keywords:** aortic stenosis, meta-analysis, imaging biomarker, myocardial fibrosis, remodeling, calcification

## Abstract

**Background:** Detecting among patients with aortic stenosis (AS) those who are likely to rapidly progress, yet potentially benefiting from prophylactic aortic valve replacement, is needed for improved patient care. The objective of this study was to evaluate the role of imaging biomarkers in predicting the progression to clinical symptoms and death in patients with AS.

**Methods:** We searched the Pubmed and the International Clinical Trials Registry Platform databases for studies including patients with AS, and investigating imaging techniques, published in any language until Jan 1, 2018. Eligible sets of data include effect of imaging biomarkers relative to: (1) Overall mortality, (2) Cardiac mortality, and (3) Overall events (Symptom onset and Major Adverse Cardiovascular Events). Meta-analysis was used to examine associations between the imaging biomarkers and outcomes of AS using Random Effect models.

**Results:** Eight studies and 1,639 patients were included after systematic review. Four studies investigated aortic valve calcification (AVC) whereas the remaining investigated biomarkers provided by cardiac magnetic resonance (CMR). Four articles investigated the presence of midwall fibrosis on late-gadolinium enhancement imaging, three reported its extent (LGE%) and two, the myocardial extracellular volume (ECV). By decreasing strength of association, there were significant associations between cardiac mortality and LGE% [Relative Risk (RR) = 1.05, 95% Confidence Interval (CI) 1.01–1.10]; overall mortality and AVC (RR = 1.19, 95%CI: 1.05–1.36); overall events and ECV (RR = 1.68, 95%CI: 1.17–2.41); cardiac mortality and midwall fibrosis (RR = 2.88, 95%CI: 1.12–7.39).

**Conclusion:** AVC and myocardial fibrosis imaging biomarkers predict the outcomes in AS, and help understanding AS pathophysiology and setting therapeutic targets.

## Introduction

Degenerative aortic stenosis (AS) is one of the most common valvular diseases, affecting up to 6% of subjects over 75 years old in developed countries (1). AS progresses with time in such a way that the only effective treatment is surgical or transcatheter aortic valve replacement (AVR). In the recent years, it has been growingly acknowledged that AS encompasses a wide spectrum of pathways in response to the progressive obstruction of the left ventricular (LV) outflow. These include first adaptive responses such as LV concentric hypertrophy that relieves the wall stress in response to LV overload, then maladaptive responses such as myocardial ischemia and fibrosis that eventually lead to myocardial dysfunction and cardiac output failure (2, 3). The concurrent progresses in computed tomography (CT), cardiac magnetic resonance imaging (CMR) and positron emission tomography (PET) have given rise to imaging biomarkers allowing quantification of the structural remodeling of both the aortic valve and the underlying myocardium (4). On a clinical view, the current indications for AVR are severe AS (peak aortic jet velocity ≥4 m/s, mean transvalvular pressure gradient ≥40 mm Hg, aortic valve area (AVA) ≤ 1.0 cm2 or ≤ 0.6 cm^2^/m^2^) causing clinical symptoms, or a decreased LV ejection fraction (<50%) (5, 6). Nevertheless, intervening too late in the disease course (i.e., when adverse remodeling and fibrosis processes have become irreversible) is associated with poor post-operative outcomes (7). Even with severe AS, the symptoms may be difficult to unmask in aged patients, as almost one half report no symptom at the time of diagnosis (8). There is therefore a need to detect from clinical, biological, and imaging tests, patients with AS who are likely to rapidly progress to symptoms, yet potentially benefiting from AVR. Several imaging biomarkers have been or are currently being considered at different levels of evidence to stratify the risk in asymptomatic severe AS. The objective of this study was to determine which imaging biomarkers (derived from CT and CMR) were associated with the prediction of AS progression to clinical symptoms and death.

## Methods

We carried out a systematic review in accordance with the PRISMA guidelines, following a protocol in accordance with the PRISMA-P statement (9). The online free database Medline (via PubMed) was searched for eligible articles. The date of the last search was January 1, 2018. The International Clinical Trials Registry Platform was searched for ongoing studies. The literature search was performed with assistance from an experienced librarian. The search strategy combined four sets of search terms (keywords), in accordance with the “Patient-Intervention-Control-Outcome” methodology. The first set of keywords defined AS (i.e.: aortic valve stenosis…), the second defined imaging techniques (i.e.: Computed Tomography, Electron-Beam Tomography, Magnetic Resonance Imaging, Positron Emission Tomography …), the third defined remodeling processes (i.e.: calcification, hypertrophy, fibrosis, ischemia…), and the fourth defined the clinical outcomes [i.e.: death, mortality, cardiovascular events (decompensation, edema, angina), progression, onset, survival…]. All keyword searches were combined to subject heading searches when appropriate. The full search strategy is provided in the Supplementary Table 1. Only original papers, clinical trials and studies, controlled and observational trials, with available full-text in English were included. Studies were eligible if they included only adult patients with AS, and investigated at least one diagnostic imaging technique focusing on the calcific remodeling of the valve, myocardial microvascular obstruction, myocardial fibrosis. Studies that did not relate the imaging results to AS progression or mortality were not included. Reports of pilot studies describing fewer than five patients were excluded.

### Data extraction

Two reviewers with experience in cardiovascular imaging assessed in consensus all titles and abstracts for relevance and eligibility. The full text of potentially relevant articles was retrieved. If full text articles were not available, the corresponding authors were contacted. Reference lists from included articles were searched for other relevant articles. The reviewers extracted and processed the data in standardized extraction forms. Corresponding authors were contacted for additional information if data were unclear or incomplete. Items included the last name of the first author and year of publication, study design, objective, sample size, inclusion, and exclusion criteria, patient characteristics, AS grade, follow-up period, funding source, technical aspects of imaging modalities, methods of measurement, interpretation of imaging results, and quantitative imaging results, measure of effect sizes [as relative risk (RR)].

### Risk of bias assessment

Study quality and risk of bias was assessed using Cochrane Collaboration's tool for assessing risk of bias (10). Ten specific bias domains were used in the form of answering pre-specified questions about the methods reported by each study in relation to the risk domain, such that the conclusion is either “no” (“–”, indicating high risk of bias), “not reported” (“NR”, indicating unclear risk of bias), or “yes” (“+”, indicating low risk of bias).

### Statistical analysis

Three outcomes of AS were considered for this review: (1) Overall mortality, (2) Cardiac mortality, and (3) Overall events [Symptom onset and Major Adverse Cardiovascular Events (MACE)]. Meta-analyses were done using the *metagen* function from the R package *meta* (11, 12). For each study considered, measures of effect were represented as RR and its corresponding 95% CIs. Conversion of effect sizes were done using the approach of Borenstein et al. (13) where RRs were not reported directly. Biomarkers with more than one effect measures in a study were combined using fixed effect model. The strength of association of each biomarker with the outcomes were quantified by pooling the RRs provided by the original studies using either Fixed or Random effect models and the results are represented as forest plots. Statistical heterogeneity between studies was assessed using the I2 and Tau2. To investigate publication bias, funnel plots were produced in addition to the use of Egger's regression test. In order to rank biomarkers with respect to effects on outcomes, the average RRs and measures of variability were converted to odds ratios (ORs) using the formula at the Supplementary Information 1. These ORs were later on transformed to Hedges' g, a common index of effect size, as stated elsewhere (13). With the use of the normal-normal hierarchical model (NNHM), a Bayesian random effect model was implemented using the *bayesmeta* package in R for evaluating the strength of association for each biomarker with the outcome. A normal prior was used for the overall mean whereas a half student-t was used as prior for the measure of heterogeneity among the effect sizes of each biomarker. Posterior predictive *P*-values (PPPV) were computed using 1,000 Monte Carlo sampling. Then, sensitivity analysis for ranking of biomarker effects was done by fitting a consistency random effect network meta-analysis (NMA) assuming a common reference group for each biomarker effect. Hedges' g was computed for each study separately before being combined in the NMA (Supplementary Figure 1). The appropriate NMA model was conducted in OpenBugs using 2 chains with different starting values and a burn-in of 50 k after 500 k iterations. Convergence of the model was assessed using history and density plots (Supplementary Figures 3, 4). For each Marcov Chain Monte Carlo (MCMC) run, each biomarker is ranked using the absolute value of the Hedges' g. Probabilities of being the first, second and up till the last are estimated and represented on a Cumulative probability plot. These probabilities are used to estimate the surface under the cumulative rank (SUCRA) curve which determined the strength of the biomarker with the outcome. All analyses were done using R studio (R version 3.4.2) and a *p*-value of < 5% was considered statistically significant.

## Results

**Figure 1:**
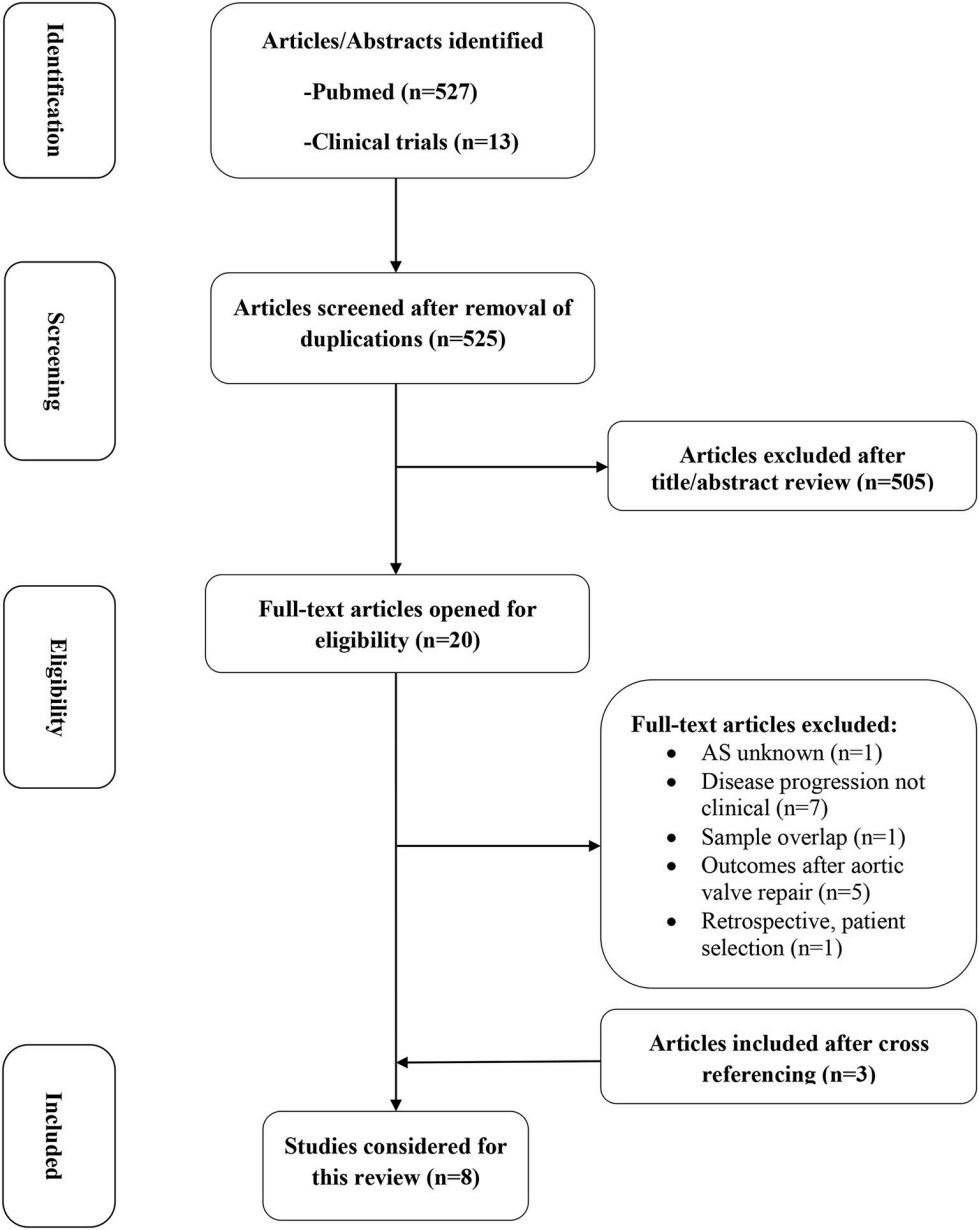
PRISMA flowchart of study selection.

The search strategy identified 540 citations (Figure 1). After screening of titles and abstracts, twenty articles were selected for full-text review. After full-text review, one study was excluded because it didn't report the patient status regarding AS upon inclusion (14). Seven studies were excluded because they used different endpoints than clinical outcome to investigate imaging results, or mixed (clinical and imaging) data to determine the outcome of AS (15–21). One other study (22) was excluded because it briefly reported the 5-year follow-up of a cohort assessed previously by Dweck et al. (23). Five studies were excluded as they report the outcomes of patients regarding imaging results, after AVR (24–28). One study was excluded because it was retrospective and evaluated only a subset of patients with low-gradient and low-flow (29). Three additional studies were found through cross-referencing (30–32). Subsequently, eight articles were included in this systematic review (Supplementary Table 2). All included studies were prospective and their sample sizes ranged from 34 to 794 patients, with a total number of 1,639 patients in this review.

The patient and AS characteristics are summarized in Table 1. Four articles investigated aortic valve calcification (AVC) with electron-beam CT (EBCT) (33) and conventional photon multislice CT (MSCT) (30, 31, 34). Four studies investigated LV myocardial fibrosis using late-gadolinium-enhancement (LGE) CMR; all investigating midwall replacement fibrosis (23, 32, 35, 36). Three of these studies investigated replacement fibrosis quantification (LGE%) (32, 35, 36), and two studies evaluated interstitial fibrosis via the extracellular volume (ECV) measurement (35, 36). Lastly, single CMR studies investigated respectively the myocardial perfusion reserve (MPR) as a marker of microvascular dysfunction in AS (36), and native (unenhanced) T1 value as a marker of myocardial fibrosis (32). Included articles reported patient cohorts from Europe, United Kingdom, USA, Canada and South Korea. The overall findings of the risk of bias assessment were: a low risk of selection attrition and outcome reporting bias, and varied risk of detection and commercial bias as the outcome adjudication blinding was nearly systematically unreported, and some investigators related to the industry in four studies (23, 32, 35, 36). All the studies recorded are of moderate-to-low risk of bias with overall quality of 50% or more (Supplementary Table 3).

**Table 1:** Patient demographics and characteristics.

**References**	**Study design**	**Follow-up (years)**	**N**	**Age (years)**	**Male *n* (%)**	**BAV (%)**	**AS grade (%)**	**Peak aortic velocity (m/s)**	**AVA (cm^2^)**	**Pmax (mmHg)**	**Pmean (mmHg)**
Messika-Zeitoun, et al. (33)	Prospective	2	100	70	58 (58%)	11	Moderate = 71 Severe = 29	2.8	1.8	NR	NR
Feuchtner, et al. (30)	Prospective	1.5	34	70.5	20 (67%)	0	NR	NR	NR	56	36
Dweck, et al. (23)	Prospective	2	143	68	97 (68%)	NR	Moderate = 40 Severe = 60	NR	0.99	70	NR
Utsunomiya, et al. (31)	Prospective	2.4	64	74	28 (44%)	0	Moderate = 55 Severe = 45	3.75	1.14	NR	29
Clavel, et al. (34)	Prospective	3.1	794	73	520 (65%)	NR	Moderate = 57 Severe = 43	3.7	1.10	NR	35
Chin, et al. (35)	Prospective	2.9	203	69	115 (69%)	NR	Mild = 17 Moderate = 22 Severe = 43	3.8	1.0	NR	35
Singh, et al. (36)	Prospective	1	174	66.2	133 (76%)	NR	Moderate = 29 Severe = 71	3.86	0.57	NR	35.4
Lee, et al. (32)	Prospective	2.3	127	68.8	63 (50%)	NR	Moderate = 38 Severe = 62	4.4	0.82	NR	48

*N, sample size; AS, aortic stenosis; BAV, bicuspid aortic valve; AVA, aortic valve area; P_Max_, maximal transvalvular pressure gradient; P_Mean_, mean transvalvular pressure gradient; NR, not reported; LGE, late-gadolinium enhancement*.

**Figure 2:**
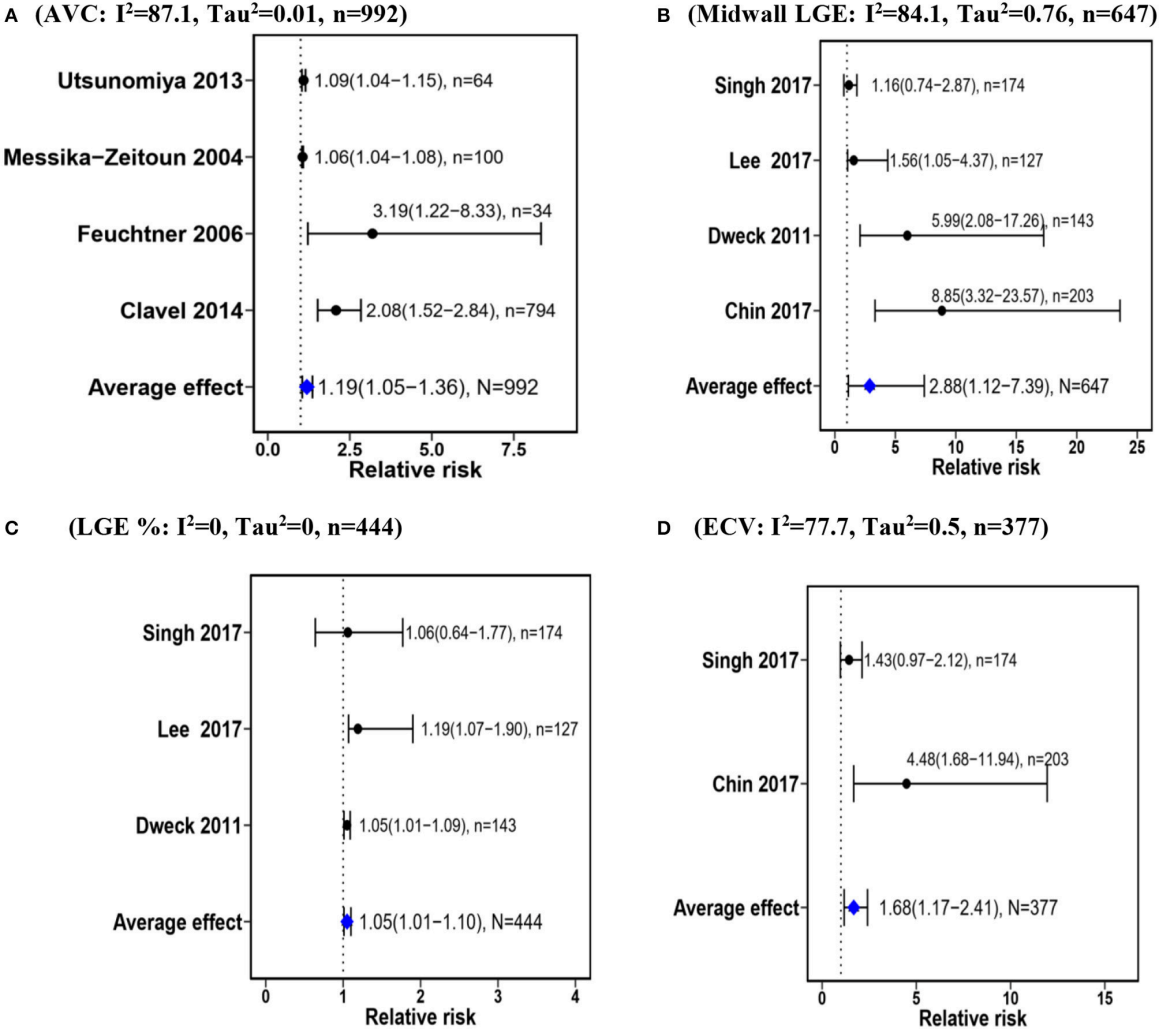
Forest plots showing the average relative risks for the strength of association between imaging biomarkers and outcomes in AS. AVC, aortic valve calcification; LGE, late gadolinium enhancement; ECV, extracellular volume. **(A)** (AVC: I^2^ = 87.1, Tau^2^ = 0.01, *n* = 992). **(B)** (Midwall LGE: I^2^ = 84.1, Tau^2^ = 0.76, *n* = 647). **(C)** (LGE %: I^2^ = 0, Tau^2^ = 0, *n* = 444). **(D)** (ECV: I^2^ = 77.7, Tau^2^ = 0.5, *n* = 377).

Meta-analyses were restricted to the biomarkers that were reported in at least 2 studies. As listed in Table 2, there were variations in study outcomes and studies with several outcomes for a biomarker was pooled into one using fixed effect model. The primary outcome was all-cause mortality in three studies (23, 34, 35), and a softer endpoint including cardiac- or AS-related mortality, MACE, AS-related symptoms or AVR in the remaining. Results from the meta-analyses confirmed significant associations between AVC and Overall mortality (RR = 1.19, 95%CI: 1.05–1.36); Midwall Fibrosis and Cardiac mortality (RR = 2.88, 95%CI: 1.12–7.39), LGE percent and Cardiac mortality (RR = 1.05, 95%CI: 1.01–1.10); ECV and Overall events (RR = 1.68, 95%CI: 1.17–2.41) (Figure 2). For all the biomarkers, higher values are associated with higher risks of having outcomes of AS, but substantial inconsistency of effect was observed for AVC, and midwall fibrosis with both **I**^2^ >75%. Because of the limited number of studies, assessment of and further correction for bias could not be sufficiently ascertained. Ranking of biomarkers in order of decreasing strength of association with the outcomes resulted in LGE% (PPPV < 0.0001) being at the top and midwall fibrosis (PPPV = 0.456) at the bottom (Table 3). Similar rankings were observed using the SUCRA values from the NMA (Supplementary Figure 2).

**Table 2:** Associations between imaging biomarkers, effect size (Variability), and outcomes in AS.

**References**	**Imaging method**	**Biomarker**	**Outcome**	**RR**	**Measure of variability (CI, SE, P)**	**LnRR**	**SE(LnRR)**
Messika-Zeitoun, et al. (33)	EBCT	AVC	OE LE ASE	1.06 1.11 1.05	1.02–1.10 1.03–1.23 1.01–1.09	0.06	0.01
Feuchtner et al. (30)	MSCT	AVC	MACE	3.18	1.64	1.16	0.49
Dweck, et al. (23)	CMR	Midwall fibrosis LGE %	OM CM OM	5.356.68 1.05	1.16–24.56 1.51–29.64 1.01–1.09	1.79 0.05	0.54 0.02
Utsunomiya, et al. (31)	MSCT	AVC	OE	1.09	1.04–1.15	0.09	0.03
Clavel, et al. (34)	MSCT	AVC (severe) AVC_density_ (severe) AVC AVC_density_	OM OM CM CM	1.75 2.44 2.14 2.28	1.04–2.92 1.37–4.37 1.08–4.45 1.11–4.95	0.73	0.16
Chin, et al. (35)	CMR	Midwall fibrosis ECV	OM	8.88 4.50		2.18 1.50	0.5 0.5
Singh, et al. (36)	CMR	LGE % ECV Midwall fibrosis MPR	OE	1.06 1.43 1.16 0.62	1.30 1.30 0.23 0.39–0.97	0.06 0.36 0.15	0.26 0.22 0.23
Lee, et al. (32)	CMR	Midwall fibrosis LGE % Native T1	OE	1.56 1.19 4.45	1.05–4.37 1.07–1.90 1.52–12.95	0.44 0.17 1.49	0.36 0.15 0.55

*AVC, aortic valve calcification; EBCT, electron-beam computed tomography; MSCT, multisclice computed tomography; LGE, late-gadolinium enhancement; ECV, extracellular volume; MACE, major adverse clinical event; OM, overall mortality; OE, overall events; LE, late events; CM, cardiac mortality; ASE, aortic stenosis related-event; CI, confidence interval; RR, relative risk; SE, standard error; Ln, neperian logarithm*.

**Table 3:** Rank of biomarkers according to posterior predictive *p*-value.

**Biomarker**	**RR**	**95 % CI**	**Hedges'g**	**95% CrI**	**PPPV**	**Rank**
AVC	1.19	1.05–1.36	0.08	0.01 to 0.13	0.010	2
Midwall fibrosis	2.88	1.12–7.39	0.11	−0.34 to 0.67	0.456	4
LGE%	1.05	1.01–1.10	0.016	0.01 to 0.02	<0.0001	1
ECV	1.68	1.17–2.41	0.15	−0.06 to 0.44	0.134	3

*AVC, aortic valve calcification; MF, midwall fibrosis; LGE, late gadolinium enhancement; ECV, extracellular volume; RR, relative risk; CI, confidence interval; CrI, credible interval; PPPV, posterior predictive p-value*.

## Discussion

This systematic review and meta-analysis showed significant associations between imaging biomarkers of aortic valve remodeling and myocardial fibrosis and clinical outcomes in patients with AS. Five years after receiving the diagnosis, approximately two-thirds of conservatively managed patients with asymptomatic AS will develop symptoms, and 75% will have either died or undergone AVR (37). During this time period, it is questionable in how far they would not exhibit raised biomarkers of poor outcome before developing clinical symptoms, altered hemodynamic or performance status. Imaging biomarkers with prognostic value in AS are often correlated with hemodynamic and clinical performance. The aortic valve calcium (AVC) score for instance is recommended in the management of patients with AS, not as a prognostic factor, but to determine the likelihood of severe AS in case of low-gradient, low-flow and preserved LVEF (6), due to its association to AS severity (16, 34). The findings of our analysis advocates for an additional prognostic use this score, as all four articles investigating AVC reported association with mortality; with an overall RR of 1.19, 95%CI: 1.05–1.36) (30, 31, 33, 34). Even though only the largest among these studies (34) introduced AVC_density_ to compensate for differences in aortic annular area, there were little measurement bias, as a highly reproducible and standardized score was systematically reported (16, 17, 38).

Similar risk stratification to AVC is expected from non-invasively assessed LV myocardial fibrosis, a fourfold potential courtesy of CMR using the effects of gadolinium-based contrast agents. These agents strongly decrease the T1 relaxation time of the tissues. As such, they can be used for track-bolus kinetics within the myocardium and assess resting and stress perfusion, thus MPR, which is potentially a marker of microvascular dysfunction (36). Gadolinium-based contrast agents distribute in the plasma and extracellular spaces, which means they do not enter normal cells. Upon equilibrium distribution (i.e., 10–15 min after injection), imaging thus figure the replacement of lost cardiomyocyte by extracellular space expansion (fibrosis), which precludes LV decompensation and arrhythmia (39–41). The presence of midwall fibrosis on T1-weighted imaging is the second biomarker derivable from contrast-enhanced CMR. Recognizing midwall fibrosis on LGE is easy and reproducible, as only requested to differentiate from post-infarct scars that classically involves the subendocardium, and amyloid, which is uncommon. In this meta-analysis, there were contradictory findings regarding the value of midwall fibrosis as a marker of clinical outcome in AS, with overall, a moderate but significant association between midwall fibrosis and cardiac mortality (RR: 2.88; 95%CI: 1.12–7.39). The relative amount of midwall fibrosis similarly accounts for prognostic value, which represents another biomarker provided by LGE. The method of quantification of midwall fibrosis depends on patient- and contrast-specific variables such as enhancement dynamics, CMR equipment and the “density” of fibrosis (42). Various cutoffs to differentiate fibrosis from the surrounding “normal” myocardium were reported across the series analyzed, including Full Width at Mid Height and Standard Deviations from the mean signal intensity histogram. Although conflicting across the series (23, 36), the percent of LGE was overall significantly associated with cardiac mortality in AS (RR:1.05; 95%CI: 1.01–1.09). A step further, assessing interstitial fibrosis necessitates more sophisticated imaging approaches aiming at establishing the T1 relaxation time mapping of the myocardium; the so-called relaxometry. Approaches using unenhanced T1 mapping (32), post-contrast T1 mapping and a mix of both have been investigated and validated against the extent of myocardial fibrosis on histology, each with its own potential advantages and limitations (43). Of these, ECV and derivatives (indexed to the body surface area) were reported in this meta-analysis. ECV represents the volume of distribution of the contrast agent within the myocardium, expressed as the difference of T1 relaxation time changes after contrast administration, corrected for the volume of distribution by using the hematocrit. ECV also showed significant prognostic effect in AS patients in our review (RR:1.68; 95%CI: 1.17–2.41).

Altogether place a special emphasis on the prognostic role of CMR in AS. However, substantial inconsistency of effect was observed for the biomarkers and the cut-offs for patient stratification varied across the cohorts. Valve calcification for instance is an active process independent from the skeletal bone calcification (44), not only associated to local factors like AS severity (15–17) or valve inflammation (45, 46), but also distant influences in relationship with classical risk factors for cardiovascular disease (47). As such, the mechanisms of initiation and progression of this biomarker are neither fully elucidated nor totally predictable. This is epitomized by the fact that females have lower AVC than males even after correction for body surface area, aortic annular area and other risk factors (48), and that severe AS with low AVC is not uncommon (49). Likewise, there is variability on a patient basis regarding both stimuli and responses to myocardial fibrosis. The investigations regarding myocardial fibrosis will need similar levels of standardization as for AVC, and a greater control for the confounders for AS-induced fibrosis or myocardial dysfunction, such as coronary artery disease or myocardial steatosis (50–52). Further studies will be needed to determine the appropriate normal value ranges of above-reported biomarkers and derivatives among subgroups by age, sex, ethnicity, and underlying risk factors and comorbidities. When accounting the current variability of these biomarkers for strength of effect using network meta-analysis, midwall fibrosis, and ECV were the weakest prognostic biomarkers (PPPV 0.456 and 0.134 respectively). This is unsurprising, as midwall fibrosis is too prevalent to make a contributive difference among patient groups, as being present in up to 62% of patients with severe AS (23–25). On the other hand, ECV and its derivatives (including unenhanced T1 values, and partition coefficient) (32, 53, 54) that are potentially reversible and sensitive to earlier adverse remodeling show considerable overlaps between normal and diseased individuals (35).

The association between imaging biomarkers and patient outcome in AS raises the question of a possible paradigm shift in the management of AS. The efficacy of a biomarker-based management as compared to the current approach that relies mainly on clinical performance need to be tested by large randomized studies. Both approaches have nevertheless the potential to be complimentary. Considering this could help refining the risk assessment in severe AS where patients with good symptom/performance status and low level of relevant imaging biomarkers being at low-risk, needing no AVR, whereas those with altered symptom/performance status and high level of the same imaging biomarkers requiring AVR. Consequently, critically evaluating the benefits of AVR in intermediate-risk groups (i.e., patients with either altered symptom/performance status or raised imaging biomarker of poor prognosis) could be a major research issue in the near future.

## Limitations

The aim of this review was to provide an overview of imaging biomarkers that could possibly predict clinical evolution in patients with AS. Our search revealed only a small number of studies, though there are other imaging biomarkers at earlier phases of their development. Some of these newer techniques use radiotracers (46) and others evaluate longitudinal or circumferential myocardial dysfunction (20, 27, 28), or wall stresses flow and deformation pattern changes (55). Our findings link imaging biomarkers with mortality, cardiac mortality or overall events. Nevertheless, AS-related events are often difficult to report and subject to bias. The proportion of patients with severe AS upon enrolment varies from 29 to 71 percent across the series (Table 2), indicating some potentially enriched cohorts, though the consecutive enrolment information missed in all but two articles (23, 33). Only one study evaluated potential selection bias via evaluation of the events that occurred >1month after enrolment (33). Most articles did not specify the blinding of the endpoint adjudicator(s). While AVR was often reported in patients who did not experience symptoms, all-cause mortality, cardiac mortality, and symptoms account for other risks than the sole severity of AS. This was underscored in the study of Clavel et al. where the survival after AVR was improved only in patients with high AVC (34).

Lastly, it should be acknowledged that: first, the prognostic value of imaging biomarkers does not necessary outperform clinical test exploring the same pathway when available. Indeed, the only study that evaluated the prognostic value of MPR as a marker of microvascular dysfunction reported a significant prediction for overall mortality (HR: 0.62; 95%CI: 0.39–0.97; *p* = 0.035), which was nevertheless not superior to that of a positive exercise testing (36). The financial burden of risk stratification using imaging biomarkers that uses sophisticated and costly imaging techniques could thus be reduced by developing more cost-efficient clinical or biological biomarkers (19, 56–59). Second, whereas the ideal biomarker for a disease should be sensitive and consistent across age, gender and ethnic groups, the current imaging biomarkers are imperfect by nature, partly due to their specificity to only one of the pathophysiological processes. Indeed, AS is a complex disease process interplaying several pathways, placing emphasis on multi-biomarker prognosis.

In conclusion, AVC and myocardial fibrosis markers are significantly associated with outcomes in AS, and have the added potential to help the understanding of AS pathophysiology and setting therapeutic targets.

## Author contributions

AN literature search, article search, data extraction, and manuscript writing. JD statistical analysis, manuscript writing, revision, and summary. LD and LS literature search and manuscript revision. CO and PL study outline, revision, edition, and summary.

### Conflict of interest statement

The authors declare that the research was conducted in the absence of any commercial or financial relationships that could be construed as a potential conflict of interest.
