# Supplementary material for: Predicting Disease Progression and Mortality in Aortic Stenosis: A Systematic Review of Imaging Biomarkers and Meta-Analysis
Source: Front Cardiovasc Med. 2018 Aug 22;5:112. doi: 10.3389/fcvm.2018.00112 (PMC6113371; doi:10.3389/fcvm.2018.00112)
Supplement: Supplementary file 1 [file Table_1.docx]

**SUPPLEMENTARY TABLES**

**Supplementary Table 1**: Search strategy sheet

| (((((((aortic OR aorta) AND valve)) AND stenosis)) AND (((((positron AND emission AND Tomogra*) OR PET)) OR ((magnetic AND resonance AND imaging) OR MRI OR CMR OR MR)) OR ((Compute* AND Tomogra*) OR CT)) OR (electron AND bean)) AND (fibrosis OR ischemia OR hypertrophy OR (calcium OR calcification OR calcific))) AND (death OR survival OR mortality OR onset OR symptoms OR events OR progression OR cardiac OR cardiovascular OR **infarct** OR decompensation OR ede?ma OR angina) |  |
| --- | --- |
| Publication year  Before January 1, 2018 | 874 items |
| Article types   - Classical Article - Clinical Study - Clinical Trial - Controlled Clinical Trial - Journal Article - Multicenter Study - Observational Study - Randomized Controlled Trial | 736 items |
| Full text availability | 631 items |
| English Language | 611 items |
| Adults (>19y) | 527 items |
