# Supplementary material for: Predicting Disease Progression and Mortality in Aortic Stenosis: A Systematic Review of Imaging Biomarkers and Meta-Analysis
Source: Front Cardiovasc Med. 2018 Aug 22;5:112. doi: 10.3389/fcvm.2018.00112 (PMC6113371; doi:10.3389/fcvm.2018.00112)
Supplement: Supplementary file 2 [file Table_2.docx]

**Supplementary Table 2**: Included studies for the review

| **Author, year** | **Title** | **Study design** | **Sample size** | **Method** | **Frequency** | **Imaging outcomes** |
| --- | --- | --- | --- | --- | --- | --- |
| Messika-Zeitoun, 2004 | Evaluation and Clinical Implications of Aortic Valve Calcification Measured by Electron-beam  Computed Tomography | Prospective cohort study | 100/44* | EBCT | 1x | AVC score (Agatston) |
| Feuchtner, 2006 | Aortic Valve Calcification as Quantified With Multislice Computed Tomography Predicts Short-term Clinical Outcome in Patients With Asymptomatic Aortic Stenosis | Prospective cohort study | 34 | MSCT | 1x | AVC score (Agatston) |
| Dweck, 2011 | Midwall fibrosis is an Independent predictor of Mortality in Patients With AorticStenosis | Prospective cohort study | 143 | CMR | 1x | -No LGE  -LV Midwall LGE  - Infarct-like LGE  - percent of LGE (FWMH) |
| Utsunomiya, 2013 | Incremental Prognostic Value of Cardiac Computed Tomography Angiography in Asymptomatic Aortic Stenosis: Significance of Aortic Valve Calcium score | Prospective cohort study | 64 | MSCT | 1x | AVC score (Agatston) |
| Clavel, 2014 | Impact of aortic valve calcification, as measured by MDCT, on survival in  patients with aortic stenosis: results of an international registry study | Prospective Multicentre cohort study | 794 | MSCT | 1x | -AVC score (Agatston)  -AVC_density_ (AVC score indexed to annular area) |
| Chin, 2017 | Myocardial Fibrosis and Cardiac Decompensation in Aortic Stenosis | Prospective Multicentre cohort study | 203/166* | CMR | 1x | -Normal  (iECV<22.5 ml/m², no LGE)  -Extracellular expansion (iECV≥22.5 ml/m², no LGE)  -Replacement fibrosis (midwall LGE) |
| Singh, 2017 | Comparison of exercise testing and CMR measured myocardial perfusion reserve for predicting outcome in asymptomatic aortic stenosis: the Prognostic Importance of MIcrovascular Dysfunction in Aortic Stenosis (PRIMID AS)  Study | Prospective Multicentre cohort study | 174 | CMR | 1x | -MPR  -Presence of LGE  -LGE percent (5SD)  -ECV |
| Lee, 2017 | Noncontrast Myocardial T1 Mapping by Cardiac Magnetic Resonance Predicts Outcome in Patients With Aortic Stenosis | Prospective cohort study | 127 | CMR | 1x | -Presence of LGE  -LGE percent (5SD)  -Native T1 |

AVC = Aortic Valve Calcification; EBCT = Electron-Beam Computed Tomography; MSCT = Multisclice Computed Tomography; CMR = Cardiac Magnetic Resonance; LV = Left Ventricular; LGE = Late-Gadolinium Enhancement; iECV = Indexed Extracellular Volume. *Denotes patients with aortic stenosis.
