# Supplementary material for: Predicting Disease Progression and Mortality in Aortic Stenosis: A Systematic Review of Imaging Biomarkers and Meta-Analysis
Source: Front Cardiovasc Med. 2018 Aug 22;5:112. doi: 10.3389/fcvm.2018.00112 (PMC6113371; doi:10.3389/fcvm.2018.00112)
Supplement: Supplementary file 3 [file Table_3.docx]

| **Author, year** | **Consecutive enrolment?** | **Inappropriate exclusions avoided?** | **All patients complete the study?** | **Selective loss to follow-up excluded?** | **Clear definition of index test** | **Clear definition of clinical outcome** | **Blinding of index test** | **Blinding of clinical outcome** | **Outcome in prespecified protocol** | **Free of commercial funding** | **Overall quality rating** |
| --- | --- | --- | --- | --- | --- | --- | --- | --- | --- | --- | --- |
| **Messika-Zeitoun, 2004** | + | + | + | + | + | + | + | NR | + | + | 9/10 |
| **Feuchtner, 2006** | NR | + | + | + | + | + | NR | NR | - | NR | 5/10 |
| **Dweck, 2011** | + | + | + | + | + | + | + | NR | + | - | 8/10 |
| **Utsunomiya, 2013** | NR | + | + | + | + | + | + | NR | - | NR | 6/10 |
| **Clavel, 2014** | NR | + | + | + | + | + | + | NR | + | - | 7/10 |
| **Chin, 2017** | NR | + | + | + | + | + | NR | NR | + | - | 6/10 |
| **Singh, 2017** | NR | + | + | + | + | + | + | NR | + | + | 8/10 |
| **Lee, 2017** | + | + | + | + | + | + | NR | NR | - | NR | 6/10 |

**Supplementary Table 3**: Risk of bias within studies

NR denotes unreported data.
