# Supplementary material for: Predicting Disease Progression and Mortality in Aortic Stenosis: A Systematic Review of Imaging Biomarkers and Meta-Analysis
Source: Front Cardiovasc Med. 2018 Aug 22;5:112. doi: 10.3389/fcvm.2018.00112 (PMC6113371; doi:10.3389/fcvm.2018.00112)
Supplement: Supplementary file 4 [file Data_Sheet_1.docx]

**SUPPLEMENTARY FIGURES**

**Supplementary Figure 1:** Network graph for the effects of biomarkers on outcomes of Aortic stenosis. n =number of studies


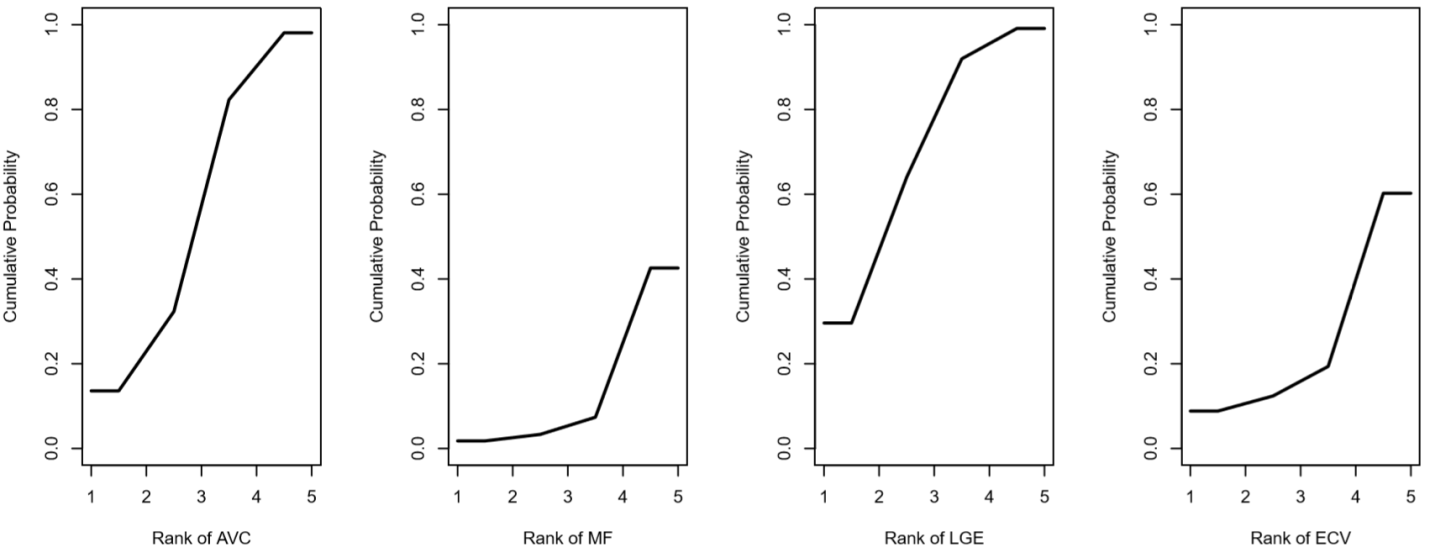


**Supplementary Figure 2:** SUCRA curve for the effect of biomarker of outcomes of AS. **AVC**: sucra=53%, **Midwall Fibrosis**: sucra=13%, **LGE%**: sucra=64%, **ECV**: sucra=23%


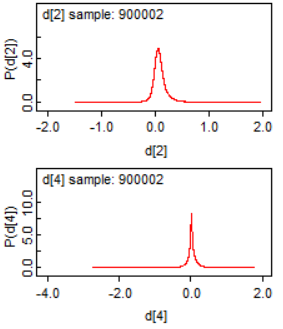

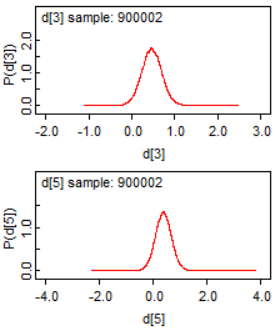


**Supplementary Figure 3:** Diagnostic density plots of Hedges’ g for each biomarker. **d2**=AVC, **d3**=Midwall fibrosis, **d4**=LGE%, **d5**=ECV


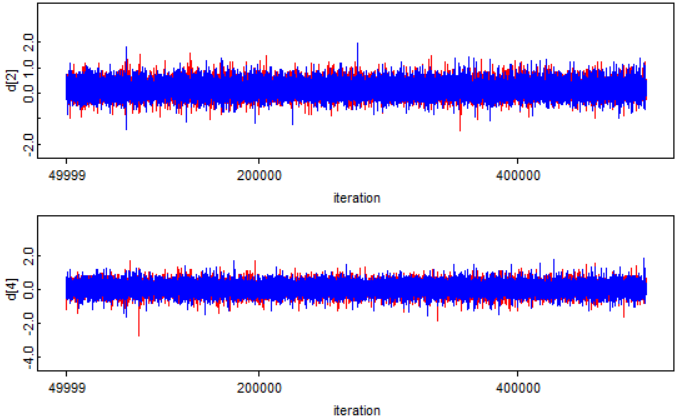

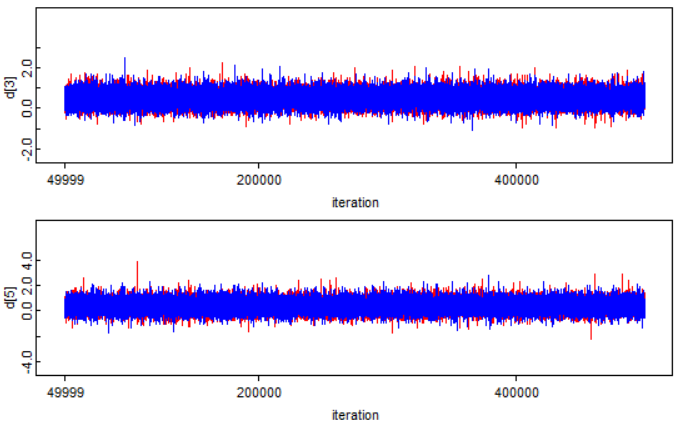


**Supplementary Figure 4:** History plots for convergence diagnostics
