# Supplementary material for: Predicting Disease Progression and Mortality in Aortic Stenosis: A Systematic Review of Imaging Biomarkers and Meta-Analysis
Source: Front Cardiovasc Med. 2018 Aug 22;5:112. doi: 10.3389/fcvm.2018.00112 (PMC6113371; doi:10.3389/fcvm.2018.00112)
Supplement: Supplementary file 5 [file Data_Sheet_2.docx]

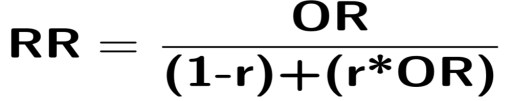


**Supplementary equation:** Formula linking OR and RR. RR=relative risk, OR=Odds ratio, r=AS rate for the population
